# What does engagement mean to participants in longitudinal cohort studies? A qualitative study

**DOI:** 10.1186/s12910-021-00648-w

**Published:** 2021-06-24

**Authors:** Cynthia A. Ochieng, Joel T. Minion, Andrew Turner, Mwenza Blell, Madeleine J. Murtagh

**Affiliations:** 1 Population Health Sciences, University of Bristol, Canynge Hall, 39 Whatley Road, Bristol, BS8 2PS UK; 2 Population Health Sciences, University of Bristol, 9th Floor, Whitefriars, Lewins Mead, Bristol, BS1 2NT UK; 3 Qualitative Research Lead, Health Technology Assessment Unit, Department of Community Health Sciences, O’Brien Institute for Public Health, University of Calgary, 3280 Hospital Dr NW, Calgary, AB T2N 4Z6 Canada; 4 School of Geography, Politics and Sociology, Newcastle University, 18-20 Windsor Terrace, Newcastle upon Tyne, NE2 4HE UK; 5 School of Social and Political Sciences, University of Glasgow, Adam Smith Building, Bute Gardens, Glasgow, G12 8RT UK

**Keywords:** Engagement, Biobank, Participant involvement, Longitudinal cohort studies, Participant experience, ALSPAC

## Abstract

**Background:**

Engagement is important within cohort studies for a number of reasons. It is argued that engaging participants within the studies they are involved in may promote their recruitment and retention within the studies. Participant input can also improve study designs, make them more acceptable for uptake by participants and aid in contextualising research communication to participants. Ultimately it is also argued that engagement needs to provide an avenue for participants to feedback to the cohort study and that this is an ethical imperative. This study sought to explore the participants’ experiences and thoughts of their engagement with their birth cohort study.

**Methods:**

Participants were recruited from the Children of the 90s (CO90s) study. Qualitative semi-structured interviews were conducted with 42 participants. The interviews were transcribed verbatim, and uploaded onto Nvivo software. They were then analysed via thematic analysis with a constant comparison technique.

**Results:**

Participants’ experiences of their engagement with CO90s were broadly based on three aspects: communication they received from CO90s, experiences of ethical conduct from CO90s and receiving rewards from CO90s. The communication received from CO90s, ranged from newsletters explaining study findings and future studies, to more personal forms like annual greeting cards posted to each participant. Ethical conduct from CO90s mainly involved participants understanding that CO90s would keep their information confidential, that it was only involved in ‘good’ ethical research and their expectation that CO90s would always prioritise participant welfare. Some of the gifts participants said they received at CO90s included toys, shopping vouchers, results from clinical tests, and time off from school to attend data collection (Focus) days. Participants also described a temporality in their engagement with CO90s and the subsequent trust they had developed for the cohort study.

**Conclusion:**

The experiences of engagement described by participants were theorized as being based on reciprocity which was sometimes overt and other times more nuanced. We further provide empirical evidence of participants’ expectation for a reciprocal interaction with their cohort study while highlighting the trust that such an interaction fosters. Our study therefore provides key insights for other cohort studies on what participants value in their interactions with their cohort studies.

## Background

Engagement is key in cohort studies [1] and is often cited as important for participant recruitment [2] and retention [3–6]. Cohort studies, particularly those of a longitudinal nature often store large amounts of data from participants, those cohorts focusing on health are increasingly adopting periodic participant biomedical sample collection and biobanking [7, 8]. Considering the detailed and often personal nature of data collected in these studies coupled with the collection and storage of bio-samples over time, such cohort studies have significant ethical implications including participant confidentiality, management of incidental findings, how to achieve informed consent and benefit sharing [6, 9, 10]. Given that participants are affected by the research from the cohort study, it is argued that there is a need for their voices to be considered by the study through engagement [11, 12]. Participant engagement is therefore necessary from an ethical perspective [13]. Additionally, because cohort studies are often funded by public funds, it is posited that they should conduct participant engagement in order to give the participants-the contributors of public funds and in whose interest the public funds should serve- an opportunity to give feedback to the study [14]. This can be viewed as a way promoting the legitimacy of studies [6, 13] particularly with regards to appropriateness- including acceptability, cultural consideration, accessibility and clarity of research protocols (including medical procedures and diagnostic tools) and documentation as well as dissemination strategies. Engagement can be understood as authentic input into research and policies of the cohort study by the public through dialogue with the cohort managers, those formulating its policies and other stakeholders [12, 15]; this in our opinion necessitates a mutual exchange and uptake of ideas. In 1996, INVOLVE was established as part of the National Institute for Health Research (NIHR), a UK government funded program to support the involvement of the public in public health, national health service and social care research; as a national advisory group it promotes public involvement in all stages of the research process [16]. However, within cohort studies involving healthy participants, engagement can sometimes be omitted from study plans, partly due to a lack of recognition of its importance and partly due to a perception that it is resource intensive (financial and time) [2, 6]. In current practice different organisations use the term engagement differentially and ambiguously [17]. Recently, studies have been conducted on cohort studies’ recruitment and retention strategies [2], however these have not reported on the participants’ experiences of their own engagement. Of the cohort studies that do report on their engagement activity, such information primarily details strategies employed to facilitate initial participant recruitment [2] or retention campaigns [4, 18]. Subsequently, older models of engagement, involving unidirectional information dissemination have been criticised for purely instrumental goals speeding product development and fostering consent among the public [19]. Engagement has been proposed as requiring authentic input into research and its policies by the public through dialogue between participants and the public with cohort managers [12, 15]- a process facilitating true partnership between participants and the cohort managers [20]. In this study, while being intrigued by these different models of engagement, we aimed to explore what authentic engagement meant for cohort study participants.

Following presentations at a workshop by Cohort and Longitudinal Studies Enhancement Resources (CLOSER) in the UK, different cohort studies presented on their activities aimed at fostering participant engagement [3]. In the publication, CLOSER describe the engagement efforts as a continuum ranging from participants having a passive role through to an active one [3]. While the publication provides a useful resource for examples of activities undertaken by cohort studies, our study goes further by providing the participants’ perspective on their engagement.

Three main conceptual underpinnings of engagement have been described in cohort studies and biobanking literature: altruism, solidarity and reciprocity. Altruism has been described as behaviour intended to benefit another even if doing so may entail some sacrifice to the actor [21]. Although participation in cohort studies is often described as being based on altruism [22], participants’ expectations such as return of research results highlight the need for a more reciprocal interaction [23]. Solidarity has been described as the manifestations of people’s willingness to carry costs (financial, emotional, social etc.) to assist others [24]. Within cohort studies, this would involve viewing participation as an act of coming together to contribute to a medical breakthrough such as fighting cancer [25].Cohort study participation is often framed as being based on both personal altruism and social solidarity [26]. Being involved in health research projects such as biobanks is often portrayed as an act of solidarity [22], much the same way as it is in donation of blood and organs for transplant [26]. Richard Titmuss described a type of gift relationship that explained how blood donation occurred in different cultural contexts [27]. Titmuss’s idea of the gift relationship have since been incorporated into explanations of cohort research participation [27–33]. Consequently, research participation and donation of samples is often formulated under the rhetoric of gifting, with the implication of it being a voluntary and free giving exercise without any expectation of reciprocity [34]. Rather than portraying the gift relationship as a reciprocal exchange, altruism and the gift are seen as non-reimbursable and unconditional [30]. An earlier conceptualisation of the gift by Mauss [35] was largely based on a communal model, which demanded reciprocation [32, 35] unlike Titmuss’s conceptualisation.

We anticipate that the participants’ perspective will provide a rich insight which will serve as useful feedback to cohort studies and enable them to focus on what participants find important. Crucially, the results presented here are also aimed at providing a data-backed and evidenced theoretical underpinning for participants’ engagement in cohort studies. The persisting ambiguity and need for theorisation and conceptualisation of participant engagement is an on-going gap in practice [17].

## Methods

The objectives of this study were to: explore cohort participants’ experiences of engagement in their cohort study, explore cohort participants’ perceptions of their engagement with their cohort study, and characterise the theoretical underpinnings of participants’ understanding of their engagement with their cohort study.

### Avon Longitudinal Study of Parents And Children/Children of the 90s

Participants within this study were recruited from the Children of the 90s study. Pregnant women in the then Avon county of England with anticipated births between 1991 and 1992 were invited to a research study [36]. The study was dubbed Children of the 90s (CO90s) or the Avon Longitudinal Study of Pregnancy and Childhood, which then became the Avon Longitudinal Study of Parents and Children-ALSPAC [36, 37]. Recruitment was through hospitals, community events and media campaigns [36] and 14,541 children were initially enrolled [38]. For clarity, this paper will focus on the experiences of the children (now adults) of the mothers who were originally recruited to the study and term them as the ‘participants’. Given that participants were born within the two-year period (1991–1992), they are all of a similar age. Since childhood, participants have contributed to CO90s in a number of ways including: regular questionnaires [39], and participating in Focus days (clinical data collection events) at which physical measurements and psychological information were gathered [37]. Sub-sets of participants are also periodically involved in smaller sub-studies for example genotyping for DNA methylation studies, genome wide association studies and research based on Mendelian randomisation [39]. Please note that the study website contains details of all the data that is available through a fully searchable data dictionary and variable search tool [40]. CO90s has a participation team that regularly communicates with participants through newsletters, greeting cards and social media. CO90s also provides opportunity for participants to be involved in the research through operational groups- the Original Cohort Advisory Panel (OCAP) and the ALSPAC Ethics and Law Committee-ALEC [41, 42]. OCAP meets six times a year and allows participants to give their feedback on engagement strategies, sub-study designs and dissemination activities [42]. CO90s participants also sit as members on the ALEC which consists of clinicians, researchers, legal experts and participants of CO90s. The ALEC reviews research ethics applications for studies intending to use ALSPAC data and either accept, reject or recommend changes to research proposals [41]. CO90s was an appropriate choice of cohort studies as they employed both unidirectional information dissemination as well as availing opportunities for participants to feedback to the cohort and input into decisions on the research conducted within the cohort.

### Study design

Qualitative research was deemed the most appropriate methodology for this study as it facilitated an in-depth understanding uncovering the processes and meanings [43] that participants held on engagement; thereby generating theory. Qualitative research enabled us to look at naturally presented language as well as experiences and the meanings derived from these [44] facilitating a thick description and understanding of the social phenomenon [43, 45] that is engagement within CO90s.

### Sampling

Purposive sampling was employed [46] in order to pick individuals with varied levels of uptake of CO90s research activities. CO90s categorises its participants into three levels based on their participation in the research activities. The calculation of a participation index (S) is based on attendance at Focus days (a) and responses to questionnaires (b) relative to the total number of Focus days (C) and total questionnaires (Q). As in [47]:

$${\text{S = }}50\left( {{\text{a}}/{\text{C}}} \right) + 50\left( {{\text{b}}/{\text{Q}}} \right).$$

This results in the three participations levels: (1) disengaged—individuals with a participation index of less than 50; (2) moderately engaged—those who have a participation index of 50–89; and (3) highly engaged—individuals who have a participation index of more than 90 [47]. Members of the OCAP and ALEC are also categorised as highly engaged. We applied purposive sampling involving the use of known characteristics on a group [44] in this case different participation levels and biological sex classification to try to obtain rich information [48] based on unique variation arising from these characteristics [49, 50]. Our sample also included some participants who had previously left the study for several years but had then decided to either return to CO90s or just to take part in our interviews. Recruitment proceeded by CO90s mailing our study information sheets to participants who then replied if they were interested in participating. Follow-up invitation letters were sent to participants who had not responded a month after their initial study invitation had been sent to them. Details of our study were also posted in the CO90s social media platforms as well as in their periodic newsletters to participants. See Table 1 for participants’ recruitment from the different participation levels in CO90s and their biological sexes recorded. In total, 42 CO90s participants were interviewed. In line with CO90s practice, participants were given a £25 cheque for participation and reimbursed for travel expenses.

**Table 1:** participants’ recruitment and characteristics

CO90s engagement level	All Participants		Male		Female	
	Invited	Interviewed	Invited	Interviewed	Invited	Interviewed
Highly engaged	140	25	41	8	99	17
Moderately engaged	301	12	187	8	114	4
Disengaged	400	5	209	2	191	3

Given that the participation groups correlate to participants uptake of research activities, it is unsurprising that their participation in our study mirrored these categories in that a higher percentage of the highly engaged participants took part in our study as compared to the moderately engaged ones, who in turn were a higher percentage than the disengaged ones.

### Data collection

Semi-structured interviews were conducted using a topic guide that had been piloted and refined for clarity. Interviewing was considered most appropriate for data collection in order to: gain insight into experiences that could not be observed, obtain their understanding of the world and the meanings they ascribed to phenomena [49], and obtain details that could only be found by exploring their experiences [51]. A topic guide was formulated and piloted on: research students of a similar age and background to the intended participants, academics with expertise in the subject and lay friends not involved in academia. The topic guide was also reviewed by the OCAP. These activities resulted in improving the clarity of the questions and order of the topics to facilitate better flow of conversation. The interviews were audio-recorded and transcribed verbatim.

### Data analysis

The transcripts were anonymised and uploaded onto Nvivo software for analysis. Analysis was based on thematic analysis [48] and constant comparison [52]. This involved an inductive search for patterns arising from the thick descriptions from the participants involving: data familiarisation, generating initial codes, seeking themes from the codes and reviewing the themes against the coded data. Through constant comparative analysis, the theories developed from the initial interviews analysed were applied to subsequent interviews to assess for consistency, and if not, changes made to attain consistency with the data [52]. Co-authors checked the codes developed alongside the raw data as a form of inter-coder agreement [53].

### Ethics

Guidance on the study design and documentation was sought from the CO90s Original Cohort Advisory Panel (OCAP) and ethical approval for the study was sought from the ALSPAC Ethics and Law Committee (ALEC). Informed consent for interview participation was obtained from participants following the recommendations of the ALSPAC Ethics and Law Committee at the time.

## Results

Through the interviews, participants described what made them feel most engaged and indeed disengaged with the study. Analysis of these descriptions resulted in an over-arching theme of participants feeling that engagement was achieved through activities that embodied a reciprocal interaction with CO90s. These ranged from subtle activities such as receiving regular communication from CO90s and CO90s adhering to ethical practice to more overt expressions like CO90s giving them rewards for participation. Their understanding of their engagement with CO90s and its embodied reciprocity also resulted in participants having high levels of trust in CO90s. Interestingly, most participants including those categorised as disengaged and moderately engaged felt they were engaged with the study. Their assessment of their engagement therefore did not just translate into the number of research activities done (as with the participation indices described above), rather their thoughts about their connection and relationship with the study. Reported below are participants’ descriptions of their engagement and the meanings they attached to these. The experiences are categorised as: communication with CO90s, experiences of ethical conduct from CO90s, rewards from CO90s, temporality of their engagement with CO90s and ultimately the trust they developed in CO90s.

### Participants receiving CO90s communication

Communication with CO90s was sometimes described more generally as engagement among some participants. This was described as a bi-directional flow of information resulting in a ‘mutually beneficial relationship’. This was expressed by participants regardless of their participation levels in CO90s. Some participants described how they engaged with CO90s and CO90s engaged with them, as such they were both engaging with each other. The perceived bi-directional engagement through communication, highlighted the reciprocal nature of their perceived engagement with CO90s.

…engagement, it’s not just a one-way thing it’s like a two-way thing– you’re both engaged in something. And it’s … usually mutually beneficial… a two-way kind of mutual goodness. (PT6, moderately engaged)

Participants describing engagement as a two-way interaction was akin to a personal relationship. They described how CO90s did not just treat them as mere data sources, instead participants felt that CO90s cultivated a relationship with them through communication. The communication included postal mail as well as telephone calls explaining research activities and establishing a rapport with them. CO90s staff were famed by participants for being very friendly and welcoming, therefore receiving a telephone call from CO90s enhanced some participants’ feelings of being wanted and appreciated by the study, further feeding into the reciprocal relational dynamic of their engagement.

Engagement…means creating that personal relationship with participants, which is crazy when you think about it, because of the number of participants in ‘Children of the ‘90s’ to have that relationship with all of them is nye on impossible, but I think ‘Children of the ‘90s’ have done it pretty well… little follow up’s that make it engagement rather than dutiful participation. (PT12, disengaged)

Participants described the communication as either informing them of research findings and upcoming research studies, or CO90s expressing appreciation to them. Participants made a connection between the reported research findings in the newsletters and their contributions to the study. This inculcated a sense of partnership with CO90s and a feeling of their importance to the study. Participants felt that CO90s reporting the research findings to them was a way of the study ‘giving back’ to the participants. This bi-directional giving highlighted a perceived reciprocal interaction with the study. This was reported by participants regardless of their participation levels within CO90s.

I expect a sort of feedback, which they are very good at doing, because I get the newsletters and they give you an update on what their research is pointing towards and what it is contributed to… that I have some idea of what my little part to play in the overall data set. (PT12, disengaged)

It [engagement] means being transparent about what’s going on and letting us know, people wanna know about when the next Focus visits are coming up and what findings have come from stuff so far and findings... linked to…things which we remember doing… I think it’s like keeping us up to date and informed mainly and there’s been some events lately as well which have been really nice…so that you can go and just find out a little bit more about what’s going on. (PT15, highly engaged)

This was further exemplified when CO90s organised other activities to provide opportunity for participants to celebrate or hear more about the research, such as when they held a summer school with lectures on their research findings for participants. These were organised when participants were young adults.

It’s about it being a two way street of information, them giving us enough to want us to continue to participate…and offering … like the summer lectures… That’s giving something back, to me, that’s what the engagement process is about, giving me the opportunity to access something as a result of my participation…I think that’s quite important.… I can see it’s a mutually beneficial relationship that we contribute and they share what they find with us and as long as, I’m interested, then they’ve got that engagement level. (PT14, highly engaged)

CO90s featuring in the local news, was also reported to enhance participants’ feelings of engagement with it. The media reports would highlight the findings from CO90s to the wider community. For participants, this not only raised their perceptions of the profile of the study, but subsequently also raised their thoughts of their importance within the study. The result was that participants were not just proud of CO90s and its successes but also proud of being a contributor to the success.

Living in Bristol, if you are watching the local news and they’ll say … “something been looked into”, nine times out of ten, if it is to do … with like healthcare, you know it’s going to be ‘Children of the ‘90s’. And I think… for me that’s really good because I can watch the news. And then I can be like, I’m part of that, I’ve helped with that. (PT8, moderately engaged)

I did feel a bit proud then because I was like “Well that’s good that my answers and everyone else’s answers has helped them to do this, this, this and this” because they talked about how they’d helped scientific organisations in America and how their information has helped provide changes in child psychology and more information on childhood vaccinations and stuff like that … I did feel quite pleased with that. (PT9, disengaged)

Participants also described how CO90s had given them a certificate for participation. This was a simple example of one way in which reciprocity or the idea of ‘giving back’ to individual participants was actioned.

they gave me a certificate once as well when I was 18 and that made me quite happy. (PT9, disengaged)

A more personal form of communication mentioned by participants was the use of greeting cards. The cards, particularly the birthday cards were seen as CO90s seeing them as individuals and commemorating each participant’s birthday gave them a sense of being special within the study. These feelings of appreciation and being special fostered feelings of being valued and being in a reciprocal relationship with CO90s.

I think…because you could say we are going out of our way and giving up our time, I think they should expect that we should want recognition for giving our time and that we should want to sort of feel involved. I think appreciation and thanks which is what they do with little birthday cards and stuff, I would say that is it just recognition and appreciation. (PT13, highly engaged)

Conversely, the less communication and contact that participants received from CO90s, the less engaged they felt. For example, CO90s began to reduce its contact with participants during adolescence, the Focus days, questionnaires and newsletters reduced in frequency thereby resulting in participants feeling less engaged with the study.

I suppose maybe a bit recently just because there haven’t been that many questionnaires coming through but …it’s not – I don’t feel it’s for me to go to them and say “Can you give me a questionnaire, please?” So no, I just wait– and that’s fine. (PT24, moderately engaged)

One integral way in which some participants were engaged in CO90s was through being involved in operational groups such as the ALEC and the OCAP. The OCAP, originally the Teenage Advisory Panel (TAP) was formed by CO90s for participants during their teenage years. These operational groups provide an important opportunity for participants to be involved in the governance of CO90s and in so doing be able to feedback to the study as well as have some input and influence within CO90s. CO90s giving the participants the opportunities within OCAP and ALEC embodies reciprocity by giving participants that governance role and ability to comment on the research conducted in the cohort and ways in which findings are disseminated. Members of the operational groups that were sampled all highlighted the great privilege they felt to be able to feedback to CO90s in these roles. They also acknowledged that while they may not have had the specialist research knowledge to comment on technical aspects of studies, they were the research participants and could provide insight into what they felt was appropriate and/or burdensome for data collection, as well as how to alter communication to better suit them (data depicting this is not shared here due to the risk of identifiability within the quotes).

### Participants experiences of ethical conduct from CO90s

Other than the communication described above, participants also highlighted that they thought that CO90s adhered to ethical practice and that this was an expression of the study’s value for and commitment to participants and therefore an act of reciprocity. The ethical principles often cited were: confidentiality, data security and prioritising participants’ welfare. The following is a further description of these as depicted in the interviews.

Confidentiality was universally expected among the participants and seen as an aspect of CO90s professionalism and commitment to participants. Participants described how they had revealed personal information to CO90s through the years, information that they had not shared even with their parents, they were confident that CO90s would never break confidentiality unless for safe-guarding reasons. This demonstrated the unique relationship that participants perceived that they had with CO90s, a relationship where they could trust CO90s to maintain their confidentiality while at the same time exercising a duty of care if there ever was a safe-guarding concern. Participants therefore felt safe enough to be honest during data collection and know that CO90s would reciprocate in their best interest.

I think there was probably things that I told researchers that I never would have told my parents. So … I expected that they wouldn’t turn round and go and tell my parents those things, which obviously they didn’t because of confidentiality. (PT18, highly engaged)

Some participants reported that they were happy for CO90s to be in control of the data as long as they exercised confidentiality, however if the confidentiality was breached then their relationship with CO90s would change. This demonstrated that some participants attached some conditionality to their donated samples and data. These participants gave their data and samples to CO90s on condition that CO90s protected the data and maintained their confidentiality. As a result, if CO90s failed to adhere to these ethical expectations, some participants said their relationship with the study would be affected and they may reconsider contributing to the study in future. In the interviews it was found that the conditionality attached by participants to their data and samples are partly based on expectations held by participants. Such expectations may be self-formed however if broken the participant-cohort study dynamic would be negatively affected.

I think the data they get from us is theirs, and as long as they keep it confidential I’m happy with what they decide is best to be done with it, I’m happy for them to use their judgement and do what they think’s necessary…confidentiality’s the only important thing, because that’s one of their main promise to… all the participants if they lost that, if they had a confidentiality leak, I think everyone would feel quite uncomfortable. (PT20, highly engaged)

Just as participants’ spoke of confidentiality as CO90s commitment to them, they also described data security as an expectation. Reciprocity is seen here in the way that participants expected, in return for their involvement that their data would be stored securely. Participants’ expectation of data security and indeed confidentially was borne from their past experiences with CO90s. Some participants also saw CO90s as a custodian of their data, not only protecting it from breaches but also being selective on who has access to it. From their history with CO90s, participants had come to understand this as CO90s commitment to them and an expression of their perceived reciprocal relationship.

On a basic level I expect … all the security that they do at ‘Children of the ‘90s’ for data and also most importantly I think the assessment they do of people like yourself doing research with the data…they have to actually go through the process of checking they are verified and trustworthy. (PT12, disengaged)

Among all the participants, the belief that CO90s did ‘good research which helped other people’ enhanced their engagement with CO90s. The idea that CO90s was helping others introduced a new aspect to the reciprocity within the relationship. In this case participants were ‘helping’ CO90s by giving their data and samples, CO90s was then ‘helping’ other people in society through their research findings. CO90s therefore reciprocated participants’ participation more widely through their findings for societal benefit and public good. An example is when looking at the requirement for venepuncture, although a lot of the participants were not fond of syringes, they were willing to endure them because they knew that it was for a good cause.

Of course there’s the blood samples, which I didn’t look forward to but it’s just the once occasional sort of thing which, I put up with to help it because, you knew it was doing good. (PT22, highly engaged)

Another way in which participants felt that CO90s kept them engaged and reciprocated their participation was through their confidence that CO90s would always prioritise their welfare. Again, this was in part due to their precedence with CO90s, having always felt that their welfare would be prioritised by the study including actions from CO90s to cater to participants’ needs. For example, participants described how CO90s had provided breakfast after fasting studies, and looked after their wellbeing during the Focus days.

I feel like if they … do something like the fasting, they always make sure that there is something for you here, like you don’t have to go out of your way to make sure that you buy something or bring something in, I think it’s always there for you and that’s really nice. (PT7, moderately engaged)

Even though study ethics are formally reiterated to research participants through both written and verbal communication, it is notable that participants in this study had high levels of confidence in CO90s commitment to research ethics and integrity. They perceived CO90s ethics as the study’s commitment to participants and inspired their trust in CO90s. This trust enhanced perceptions of being in a reciprocal relationship with the study. Other than the communication and ethical conduct described above, a more overt reciprocity described by participants was in CO90s giving them rewards for participation as described below.

### Participants receiving rewards from CO90s

In the interviews, engagement was sometimes described as CO90s giving the participants something as a result of participation.

I think, to be engaged, that you have to be getting something out of it yourself. (PT6, moderately engaged)

Participants always received a reward for their involvement in the study either through receiving a voucher, toy, time off from school when they were younger or even return of research results. Receiving a gift made participants feel valued and motivated them to continue participating in the research. All participants in our study made reference to the vouchers (as they got older) and toys (when they were younger) that they received from CO90s in reciprocation for their involvement. Interestingly, the significance of the vouchers and toys lay not solely on their monetary value but rather on their relational significance. In a sense it was not the amount of money that mattered rather it was the idea that they had been offered the money (voucher) in the first place. This was a key way in which participants were engaged with the study and was described by participants from all the participation levels.

Well, I suppose the term ‘engagement’ is two parties coming together so, I suppose, engagement with the Children of the ’90s is me going to the organisation and putting something into it, and what comes out of it is information that is used as a whole and data and, when I was younger, the vouchers. (PT1, moderately engaged)

It was always good because…as a kid we always got the reward at the end … we just had to answer a few questions, do a few tests and ... We’d always get something. We’d always get some treats at the end and I think as we got a bit older they would give us money at the end... (PT2, moderately engaged)

I more enjoyed the fact that I was turning up and after about three hours they’d give me a £20 voucher for Amazon, or something that I could go and spend. (PT3, disengaged)

When I was in secondary school I did expect some money back for me spending my time here. (PT4, highly engaged)

Memories of receiving the body scans and clinical measurements were seen as ways in which CO90s reciprocated to participants; as participants gave data and samples to the study in return CO90s gave them feedback on the clinical tests. Ironically, although participants knew that the tests were not a health check, they still anticipated CO90s to act if they discovered any anomalies. Part of the reason why participants in CO90s expected to receive incidental findings is because in the past some had been given feedback of diagnostic importance.

The way I always looked at it was that I’ll be getting…not free … they’ll probably give us relatively frequent health checks so I’m thinking… I’ll probably get … a lot of free information about myself that … many people might have to pay for. (PT2, moderately engaged)

In regards to the commitment, I was impressed the fact that they said my eyesight was poor. I know eventually … I would’ve realised and clocked on that I needed glasses. But, in regards to that I do, still to this day hold them, almost hands off and say “well thanks very much for that, you did point that out to me”. (PT8, moderately engaged)

Participants came to associate attending the Focus day with a day off from school. The opportunity to miss school not only motivated participants, it also facilitated the children being able to attend the Focus days. Given that CO90s was conducted within the former Avon county, because of the regional popularity and informal endorsement, many of the local schools allowed the children to be absent for the CO90s Focus days. This highlights the importance of the communities’ endorsement and involvement in population studies such as CO90s. CO90s having those arrangements with schools which permitted the participants to miss school was another example of how the study tried to not only facilitate but also reciprocate participants for their involvement.

I used to really look forward to them because it meant a day off school, and my mum and I… we were quite cheeky with it, because my mum would put me in for an early morning clinic and then we’d be finished at maybe lunchtime, and then… I wouldn’t go back to school … I’d have the rest of the day with my mum (PT5, highly engaged).

I think Children of the 90’s as a kid, you’re always very excited about it… obviously I never really quite understood what it was when I was a kid to me it was just a day off school it was quite fun because anything’s better than school. (PT9, disengaged)

The fact that CO90s was located in and around Bristol also meant that many participants had classmates in CO90s and many schools recognised CO90s as a valid venture. Having other schoolmates in CO90s enhanced the impression of CO90s as trustworthy. It also normalised CO90s and embedded the study in participants’ everyday lived experiences. Having CO90s as part of their life histories fed into their feelings of trust and indeed relationship with it.

Everyone at School was part of it.( PT27, moderately engaged)

In the school setting it wasn’t strange at all, because everyone in my primary school class was a part of the study. Well, not everyone but like enough for it not to be strange when someone was saying, “oh, so-and-so’s at Children of the 90s' today; that’s why they’re not in.” So … because everybody just, knew about it or was a part of it. (PT5, highly engaged)

Many participants felt most engaged when they regularly attended the Focus days. These events offered many avenues to maintain a feeling of engagement. Participants described the experience of CO90s Focus days as fun and engaging. This teases the idea that the Focus days were tailored to the children’s interests and collected data in an age appropriate and entertaining manner. Making the sessions interesting to the participants was another example of how reciprocity can be achieved through designing data collection in line with the interests and needs of participants.

I really enjoyed it – just everything…it was really interesting to do it all (PT11, moderately engaged)

Really looked forward to it.. when I came it was always …because you get to do quite a lot of cool things like there was scanning and, they scan the body and you get to keep the picture and you got little booklets as well that shows you your height, your weight. So you could keep that information (PT7, moderately engaged)

Some participants revealed that the Focus days made them feel part of CO90s. Being in the physical premises of the study, watching the data collection and being able to see the posters bearing previous research results made the participants feel really engaged. One could argue that inviting the participants into the research premises, was a way that CO90s allowed them into an exclusive research world in reciprocation for their involvement.

I think there were times when I felt really engaged like I was really part of the ‘Children of the ‘90s’ was when you used to go to the Focus visits. Because you do so much, and you’d know that somewhere along the lines, it was really being used for the benefit of other people…I think that’s when you really feel the most engaged because… you do the questionnaires, you do the ones online but when you go to the visits you see, you see how it runs on a day to day basis. And I think that’s the one that’s got to have the impact on everything really. I mean for me that’s when I felt, well, this is ‘Children of the ‘90s’ I’m at ‘Children of the ‘90s’ so that’s probably when I’ve felt most engaged. (PT8, moderately engaged)

Similarly, when asked to describe a time when they felt disengaged, most interview participants pointed to the time when the Focus days became less frequent when participants were teenagers.

I think there was a time when the clinic visits stopped or like just become really irregular that … I’d kind of forget that it was … that I was such a part of it, and then maybe getting a questionnaire every year just reminded me it was still going (PT5, highly engaged)

The data above shows how participants either received or felt that they received a return gift in exchange for their participation. The inclusion of a return gift embodies the concept of reciprocity. Within cohort studies although reciprocity can be actioned in the form of return benefits for research participation, as demonstrated above it can also be operationalised in any ideas that recognise and facilitate a mutually desired and appreciated exchange between the participant and the cohort study.

Given that most of the participants in this study had been in it since birth, it became important to explore the temporal aspect of their engagement with the study. Below are the findings from this.

### Participants' temporality of engagement

A sense of temporality was evident when participants described their engagement with the study over time. They described their feelings of being connected to the study in personal ways such as CO90s being ‘a lifelong study’ of themselves. This perceived personal connection with CO90s and investment in the success of CO90s hinted at how engagement in cohort studies can be perceived by participants as an intimate interaction over time. It can be argued that this interaction and perceived commitment over time necessitates feelings of reciprocity between both parties. This then promotes input from both parties as being key to the success of the project.

Isn’t this like, a lifelong study of me, so they do have a, commitment to … I would like to see it through. I would like to keep doing it… cause the more and more you do it, and as you get older you can start seeing, why they’re doing things, the patterns. (PT6, moderately engaged)

I was reading yesterday that they plan on, monitoring me my entire life and hopefully any children I have…my brothers –just trying to get a complete picture as possible, I would like that to happen (PT24, moderately engaged)

Most of the participants had been in CO90s since their birth. Their engagement had therefore changed and in some cases matured as they aged. It had moved from parental decision making to participants’ own. This growing act of becoming the decision maker made them feel very engaged with CO90s. Undoubtedly, engagement of this nature is complex, it not only incorporates elements of parental endorsement, but also inhabits a temporal space in the participants life. Parental endorsement on the merits of participation incorporate reciprocity as depicted earlier in highlighting the importance of the research findings (see results on communication). The temporal aspect resulted in their engagement with CO90s evolving through their lifetime and in some cases becoming a perceived maturing relationship.

I think I’ve become much more engaged with [CO90s] as I’ve got older because I understand it a lot more now. I knew that my parents had signed me up to from birth, so I was just like “Oh, it must be fine then” and just did everything…Whereas now I’m a lot more interested in it so I do it now more for a reason, to find out and to help with a big project rather than just for the sake of it. (PT21, highly engaged)

Engagement, it’s changed since I was younger. My engagement now is autonomous. It’s something I decide on my own. My parents might say, “Oh, you’ve got a letter – you ought to do it,” or that sort of thing. I find my engagement is completely on my terms…I can make the decision whether I wanna participate. (PT19, highly engaged)

As intimated above, participants described how CO90s represented an important part of their biographical past and future including their future children and other family members. This perceived connection with CO90s further revealed the complexity of their relationship with CO90s and their perceived integral part within the study’s past, present and future.

We’ve got COCO’s now, the Children of the Children of the 90’s so we are now a third generation and we are stretching it out to grandparents… the resource that we have is unlike anything else in the UK so I think my expectation is that they keep going with it because if it just stopped, it would suck because you have this huge wealth of data of everyone up to they turn 21 but if they just cut off tomorrow them you can’t really track it through fully. (PT26, highly engaged)

### Perceptions of engagement among the ‘disengaged’

When asked about their engagement, participants who were categorised as disengaged revealed that their own perception of their engagement level was not one of disengagement. This showed that these participants understood engagement to mean something other than just the number of research activities they had participated in. For them engagement was an interaction involving the study giving them information to understand the research and the opportunity to decide to do the research activities.

I think I’m quite highly engaged now… I am engaged because I know about, like, all the research they’ve done and I’ve seen them in the news as well, as you know, just on their website and things like that. (PT28, disengaged)

how do you rate yourself in terms of engagement do you think?

Probably just above average, obviously since I was eighteen because when I was a kid I don’t think I was particularly aware of it …So I think it means…making a participant interested enough to do an interview or to do a survey or to do a longitudinal study, because they want to and because they have been given enough information about what it is contributing towards, but it makes them want to do it. That’s engagement I think, because *…* you are saying ‘this is why we are doing this, this is why we are collecting this data, it will be helpful for this, this and this, do you want to do it’, ‘yeah I’ll do that, because I am interested in this, this and this and I want to help contribute to this, this and this’. So I think engagement for me, yeah it means creating that personal relationship with participants…(PT12, disengaged)

Participants were also asked when/if they had felt disengaged from CO90s. None of the ‘disengaged’ participants interviewed stated that they had ever felt disengaged from CO90s. From their responses engagement in these instances was seen as the continued flow of information from CO90s and in some instances the opportunity for the participants to also communicate with CO90s.

have you ever felt somewhat or completely disengaged from Children of the 90’s?

No they send out letters they still... when I came back from [abroad] this time I was like “I will send them and eventually I did ring someone” so yeah, I’ve never felt completely disengaged because otherwise I wouldn’t have felt that nagging feeling like so ring them because they want to know about your blood, (PT9, disengaged)

somewhat or completely disengaged? No. [pause] No; I mean, no; but you forget about them and they keep popping up, but I guess I feel more involved than engaged at the moment. (PT3, disengaged)

The implications of this was that participants felt that they were free to seek out CO90s research activities at their own convenience and that this was a characteristic of their unique relationship with the study.

I think that’s you know as adults you’re gonna be more likely to be proactive and you’re more likely to say “I am in Bristol, I am free”…Is there anything I can do? Is there anything I can take part in? Is there any way I can help?That’s pretty much what I did, yeah because basically so I had a whole bunch of letters about the brain scan – the Cardiff brain scan when I came back so I rang them up about that …And then no one did get back to me so I rang them up then– I think I got this letter so I rang them up again and I ended up speaking to you. Because that was the thing I just – yeah, I just felt that it’s just that you know it’s just the easiest way – I did get the letter through about this so I did read it and then think “Well I’ll ring them up”. (PT9, disengaged)

I called Children of the ’90s to see when they were next doing a focus group, cause .… there hasn’t been a focus group since we were 17, and they said they were planning on doing one when we were 24 or 25,… I’ve spoken to them recently about what kind of things they are doing, and … they still focus on the genetic side of things. I think … they seem to be focusing on the … the brain and the heart...I called them. (PT28, disengaged)

Other than seeking CO90s research, another implication of participants having their own perception of their engagement was their understanding of dissenting to research activities in CO90s. The freedom to decline/dissent research activity impacts on their participation index in CO90s records, however participants are not aware of their own participation indices. Interestingly, participants viewed their freedom to decline as part of their engagement with CO90s. They felt that being able to decline research activities in CO90s was part of the reason they were happy to be a part of the study and why they felt they had a good relationship with CO90s.

I think – they’re very good like that like for example I’ve unintentionally decline many things over the last five years just because I’ve not been in Bristol and they’ll send their follow letters and – but I think they are very– they’re not very pushy and they are very polite and I think that’s good. Like I just didn’t –send the questionnaire back and that was it. Or rather I sent – because it was a questionnaire of two parts so I just sent some of the questionnaire back and not the other part and that was it. Or you can just skip questions like it’s very easy to not do stuff for Children of the 90’s they’re very polite like that. (PT9, disengaged)

I gave permission for sort of, you know, the health records … I don’t have any criminal convictions, but I didn’t give permission for that anyway. I thought that was a bit … I don’t know. I gave permission for education and I did give permission for the employment bit, but not certain bits within the employment bit – so, like wages and tax and things like that… I mean, they’re fine with people leaving and coming back or not wanting to do the focus groups or not wanting to sort of do the questionnaire and things, so they are quite tolerant of people who, you know, don’t want to partake in everything, and they’re sort of very understanding of different circumstances as well. (PT28, disengaged)

In speaking about their engagement with CO90s, participants seemed to have a lot of trust in the study. This was inherent in their descriptions of their relationship and anticipated reciprocity there-in. Below is an exploration of the role of their trust on their engagement with CO90s.

### The role of trust in engagement

Participants displayed high levels of trust in CO90s. This was demonstrated in a number of ways including their confidence in what CO90s would and would not do to them. From the descriptions above, being involved in CO90s all their lives, and having confidence in their relationship with CO90s and indeed their perceived reciprocity cultivated strong feelings of trust in the study. Additionally, because many participants had been born into the research project, their mothers seemingly already had high levels of trust in CO90s. Mothers being involved with CO90s provided a sense of confidence in CO90s, as well as an atmosphere of security and safety within the research.

Mum would say “Oh, no it’s fine you don’t have to answer a question if you don’t want to but it’s safe to…explain that it’s safe to talk to these people so you know I trusted them as a child. (PT25, highly engaged)

Subsequently, the participant-CO90s relationship was a complex tripartite relationship involving negotiations between the participant, their parent and CO90s. This echoes findings from a narrative review on paediatric cohort studies similar to CO90s[54]. CO90s continued to recruit participants’ family members (such as siblings and grandparents) which further promoted familial trust in CO90s.

Trust was also sometimes enhanced by the institutional affiliations of CO90s. Some participants had high levels of trust in CO90s partly because CO90s was associated with the University of Bristol, because it did medical research (and hence linked it with clinicians) and because it was perceived to be a non-profit organisation.

I assume it’s [CO90’s] non-profit and I know it’s academia as well…that’s just my personal attitude towards academia and non-profit organisations in general that I kind of believe, I do trust them because, there’s no profit in asking someone like myself about their life but if it helps science then I think that’s a good thing. (PT9, disengaged)

You kind of like trust Children of the ‘90’s because it’s always been there … you’re putting their trust on them to be fair and ethical and understanding and not do the wrong thing as such say just – like a doctor…you trust them to do the right thing. (PT18, highly engaged)

I see all the people that work in the research as medical doctors and so there isn’t any information that I wouldn’t share with the medical doctor. So there’s nothing that I’d be, like, they shouldn’t be asking that. I’m open to whatever they’d be interested in talking about. (PT14, highly engaged)

This section has reported on the trust that participants had in CO90s. This trust fostered a perceived relationship with the study based on expectations of both overt and nuanced reciprocity.

## Discussion

From the interviews, engagement appeared to be a concept experienced and impacted by different activities of CO90s. As described above, these activities ranged from nuanced expressions of engagement such as adherence to ethical conduct and communication with CO90s, to more overt activities like CO90s giving participants gifts. Participants’ engagement followed a complex perceived reciprocal relationship over time and resulted is significant levels of trust in the study. It is thought that authentic engagement in different cultural contexts involves mutual respect and requires trust in the biobank [55–57]. Our findings here report on the participants’ experiences and thoughts on how CO90s engaged with them and the resultant relationship and trust they developed for it. This kind of trust that participants develop for their longitudinal cohort studies has been mentioned elsewhere [58] including in other studies [59] such as the Irish Longitudinal Study of Ageing (TILDA) which reported that their participants felt they had a bond with the study and a special rapport with data collectors and that this impacted on participant retention [60]. Consistent with previous writings [30] we have evidenced here that cohort study participation should not be considered a unidirectional activity, the interviews reported here demonstrate that the perceived reciprocity contributed to sustaining the long-term commitment required for a longitudinal study. This has also been posited elsewhere [61, 62] and the results of Mathie et al. [63] in their study with PPI members found that their involvement in research was also based on a reciprocal relationship.

At the heart of the findings here is the role of relational precedence which argues that participants negotiated their relationship with cohort studies based on their past experiences of it [64]. Participants in this study had formulated their expectations of reciprocity from CO90s, based on their past experiences with the cohort study. Ma’n and Knoppers [65] also argued that research participation necessitates reciprocity as a way of expressing the value of the exchange and as a recognition of the sociality of humans in giving and receiving benefits. However, research participation has often been portrayed as gifting with particular emphasis of altruism and solidarity with little or no mention of reciprocity [32], based on Titmuss’s conceptualisation of the Gift Relationship [66]. Through this study we have presented the participants’ voice regarding their engagement, based on this we believe that if engagement in cohort studies is to be understood within the discourse of giving, there needs to be a re-examination of its application of Titmuss’s gift relationship [67]. We propose a re-thinking of a gift relationship with the incorporation of gifting as described by Mauss [35] where reciprocation is inherent within the gifting. This relationality, reciprocity and sociality is what makes the data presented here comparable with gifting [67] in Maussian terms. It is also important to note as demonstrated by the data above, that the application of reciprocity in cohort studies doesn’t always involve tangible rewards for research participation, sometimes it can be more nuanced and relational [68]. Our findings also provide empirical evidence for Hobbs et al. [34] proposal that reciprocity could cultivate an on-going collaboratory relationship between research participants, cohort studies and the wider society, as well as giving the participants a perception of control and value within the cohort study. Through our results, we can also affirm Shippee et al. [69] and Zawati and Lang [70] who propose that cohort studies such as biobanks need to cultivate bidirectional relationships requiring collaboration between research studies and participants underpinned by principles of reciprocity.

### Limitations

Due to stringent data protection protocols, the invitation to take part in this study was conducted by CO90s. As such all participants had to respond to CO90s to show their interest in being interviewed. As such participants whose addresses had changed from those in the CO90s records and who did not read the CO90s social media pages or newsletters may not have received their invitation to our study. It is also probable that some participants particularly those classified as either disengaged or moderately engaged may not have wanted to speak to us about CO90s.

## Conclusion

From the participants’ descriptions engagement was achieved through activities that embodied a reciprocal interaction with CO90s. These ranged from subtle activities such as receiving regular communication from CO90s and CO90s adhering to ethical practice to more overt expressions like CO90s giving them rewards for participation. Their understanding of their engagement with CO90s and its embodied reciprocity also resulted in participants having high levels of trust in CO90s. This perceived reciprocity aligns well with Maussian conception of gifting where reciprocation is inherent as opposed to Titmuss’s gift relationship which is often applied to highlight participant altruism and solidarity. This study is important as it provides empirical evidence of participants’ expectation for a reciprocal interaction with their cohort study while highlighting the trust that such an interaction fosters. Our study therefore provides key insights for other cohort studies to understand what participants value in their interactions with their cohort studies. Additionally, through the participants’ voices, our study has highlighted examples of how reciprocity can be practically actioned in a way that is meaningful to participants.

## Data Availability

The datasets generated and/or analysed during the current study are not publicly available due to the risk of identifying participants from the transcripts. However, they may be made available from the corresponding author on reasonable request.

## Abbreviations

NIHR: National Institute for Health Research
CLOSER: Cohort and longitudinal studies enhancement resources
CO90s: Children of the 90s
ALSPAC: Avon longitudinal study of parents and children
OCAP: Original cohort advisory panel
ALEC: ALSPAC ethics and law committee
